# Left Extended Hemihepatectomy With Preservation of Large Inferior Right Hepatic Vein: A Case Report

**DOI:** 10.1155/1999/24710

**Published:** 1999-07

**Authors:** M. L. Texler, G. G. Jamieson, G. J. Maddern

**Affiliations:** 1 The Department of Surgery The Queen Elizabeth Hospital Woodville Road Woodville South South Australia 5011 Australia; 2 Department of Surgery Royal Adelaide Hospital The University of Adelaide Australia

## Abstract

For hepatic function to be preserved after an extended hemihepatectomy adequate venous drainage of the remaining liver is required. Most metastases close to the confluence of the superior hepatic veins are considered unresectable because hepatic venous outflow after resection would be compromised. In 10–25% of people, the inferior right hepatic vein is of large calibre. Thus the superior hepatic veins may be sacrificed and hepatic function preserved if a large inferior right hepatic vein is present.

A patient with involvement of segments 2, 4 and 8 by metastatic colorectal cancer is presented. This patient had a large inferior right hepatic vein, and so was able to undergo an extended left hemihepatectomy with ligation of all superior hepatic veins. Subsequent quality of life was maintained.

This case illustrates that an ‘unresectable’ hepatic lesion can be actually resectable if an alternative venous drainage is present. A pre-operative search for a prominent inferior right hepatic vein by ultrasound, computerised tomography, or even magnetic resonance imaging should be considered in these cases.


*HPB Surgery*, 1999, Vol. 11, pp. 265-270
Reprints available directly from the publisher
Photocopying permitted by license only

© 1999 OPA (Overseas Publishers Association) N.V.
Published by license under
the Harwood Academic Publishers imprint,
part of The Gordon and Breach Publishing Group.
Printed in Malaysia.

### *Case Report*

### Left Extended Hemihepatectomy with Preservation of Large Inferior Right Hepatic Vein: A Case Report

M. L. TEXLER[a], G. G. JAMIESON[b] and G. J. MADDERN[a,*]

[a] *The Department of Surgery, The Queen Elizabeth Hospital, Woodville Road, Woodville South, 5011, South Australia, Australia;*
[b] *Department of Surgery, Royal Adelaide Hospital, The University of Adelaide, Australia*

*(Received 5 November 1997; In final form 1 July 1998)*

**For hepatic function to be preserved after an extended hemihepatectomy adequate venous drainage of the remaining liver is required. Most metastases close to the confluence of the superior hepatic veins are considered unresectable because hepatic venous outflow after resection would be compromised. In 10–25% of people, the inferior right hepatic vein is of large calibre. Thus the superior hepatic veins may be sacrificed and hepatic function preserved if a large inferior right hepatic vein is present.**

**A patient with involvement of segments 2, 4 and 8 by metastatic colorectal cancer is presented. This patient had a large inferior right hepatic vein, and so was able to undergo an extended left hemihepatectomy with ligation of all superior hepatic veins. Subsequent quality of life was maintained.**

**This case illustrates that an 'unresectable' hepatic lesion can be actually resectable if an alternative venous drainage is present. A pre-operative search for a prominent inferior right hepatic vein by ultrasound, computerised tomography, or even magnetic resonance imaging should be considered in these cases.**

*Keywords:* Hepatectomy, hepatic veins, radiography

### INTRODUCTION

Hepatic resection has been advocated for the removal of colorectal hepatic metastasis. Tumour free survival at five years post resection of colorectal hepatic metastases has been calculated as 34% [1]. Five, 10 and 20 year survival has been calculated as 40%, 24% and 18% respectively *versus* 0% for patients with non resected colorectal hepatic metastases [2, 3]. Hepatic resection has been carried out since the end of last century [4]. Early attempts at hepatic resection were compounded by: inaccessibility of the liver, lack of understanding of hepatic physiology, uncontrollable haemorrhage and infection [5]. In 1952, the first successful extended right hemihepatectomy was accomplished [6]. In 1957, Claude Couinaud published a seminal work; Le Foie: Études Anatomiques et Chirurgicales (The Liver: Ana-

*Corresponding author. Tel.: 08 8222 6000, Fax: 08 8222 6028, e-mail: gmaddern@medicine.adelaide.edu.au tomical and Surgical Studies) [7]. This work described the liver as being composed of two livers (a left liver and a right liver). Each liver containing two sectors and each sector possessing two segments (with one exception), giving eight functionally separate segments [6]. Each segment receives its own portal vein, hepatic artery and bile duct contained within a sheath (the Glissonian sheath). Relatively bloodless anatomic hepatic segmentectomies can be achieved by isolating and clamping the sheaths to segments to be resected (the posterior intrahepatic approach) [6].

There are three major hepatic veins located superiorly: left, middle and right hepatic veins [6, 8]. On the right side of the liver there are variable numbers of accessory veins. The right middle and inferior right hepatic veins are constant but normally of small calibre [8]. They drain segment 6 predominantly, but may include drainage from segment 5 [8]. In about 10–25% the inferior right vein can be quite large with a calibre exceeding 0.5 cm [6–10]. In 3% of one study, the inferior right vein was thicker than the right hepatic vein (inferior right hepatic vein dominance) [8]. This is clinically important, because the postero-inferior area of the right lobe (segments 5 and 6) can be preserved along with the hypertrophic inferior right hepatic vein, even if the right hepatic vein is resected [9]. Venous drainage from the liver needs to be intact post resection to maintain hepatic function and in many cases, a liver lesion cannot be resected due to the involvement of hepatic veins within the lesion. However, successful resections sacrificing the right hepatic vein and sparing the inferior right hepatic vein have been reported [10–12]. Following is a report of such a technique.

### CASE REPORT

The patient was a 73 year old female who presented with nausea and abdominal pain. Three months earlier, she underwent a right hemicolectomy with anastomosis for moderately differentiated Duke's C adenocarcinoma of the colon. No detectable liver metastases were noted at that time.

During the current admission, investigations included: serum biochemistry, complete blood examination, coagulation profiles and computerised tomography. The computerised tomography showed a solitary, irregular, hypodense, lobulated lesion in the liver measuring 9.5 cm by 7.2 cm in maximal diameter involving the right lobe of the liver. Given the patient's previous good health, resection of this probable metastatic lesion was considered. Further definition of the vascular anatomy was required to asses the feasability of resection.

Computerised tomography arterial portography and magnetic resonance imaging showed the lesion involving segments 2, 4 and 8 of the liver. The right hepatic vein was displaced posteriorly by the tumour. Caval compression was also noted due to mass effect as well as an inferior right hepatic vein draining segments 5 and 6 (see Figs. 1 and 2). The patient had a pre-operative American Society of Anaesthetists Rating of 3. Pre-operative blood biochemistry demonstrated: albumin 39 g/L (33–50 g/L), total bilirubin 15 μmol/L (1–20 μmol/L), serum urea 7.3 mmol/L (3–7.6 mmol/L), serum alkaline phosphatase 224 i.u./dL (30–115 i.u./L), serum aspartate aminotransferase 30 i.u./L (0–45 i.u./L), serum gamma glutamyl transferase 188 i.u./L (0–60 i.u./L), serum alanine aminotransferase 24 i.u./L (0–45 i.u./L) serum lactate dehydrogenase 430 i.u./L (100–255 i.u./L), international normalised ratio 1.11 ($<2$) and activated partial thromboplastin time 28 s (25–37 s).

Resection of the tumour was as for an extended left hemi-hepatectomy [6]. A rooftop incision was used. All superior hepatic veins were ligated. The inferior right hepatic vein was present and preserved. The right main sheath was left intact and segments 5, 6 and 7 preserved. Segments 1, 2, 3, 4 and 8 were resected along with the gall bladder and margins of tumour on right side (size

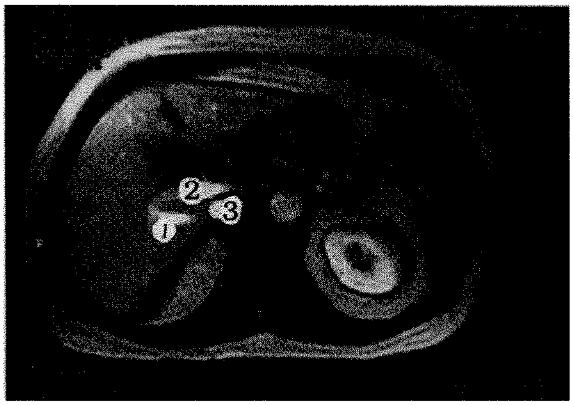

FIGURE 1 Magnetic resonance image of upper abdomen pre-operation (transverse plane). 1: inferior right hepatic vein, 2: portal vein, 3: inferior vena cava.

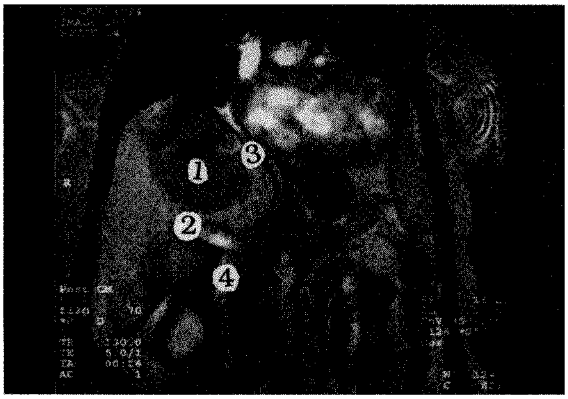

FIGURE 2 Magnetic resonance image of upper abdomen pre-operation (coronal plane). 1: tumour, 2: portal vein, 3: right hepatic vein, 4: inferior vena cava.

of specimen 110 by 80 by 50 mm, weight 730.3 gm). Oxycel gauze was applied to the vena cava. An omental mass near the previous bowel anastomosis was excised. Frozen section of this lesion was benign. Two Redivac drains were placed into the sub hepatic space and at the porta hepatis. The abdominal wall was closed with monofilament polydioxanone sutures and metal clips to skin. Anaesthetic time was 6 hours 35 minutes, with an operative time of 5 hours 45 minutes. Calculated intra-operative blood loss was 7500 mL, requiring 13 units of blood and 2 units of fresh frozen plasma.

Histopathology of the hepatic resection showed metastatic adeno-carcinoma from bowel. Post operative stay in an intensive care unit was 6 days, followed by 16 days in a high dependency unit. On day 6 post resection, a chest drain was inserted to drain a left pleural effusion. On day 28 post resection a right pleural effusion was treated conservatively. On day 29 post resection, a colo-cutaneous fistula appeared, believed to be caused by ischaemia near the old anastomosis secondary to the omental resection. The fistula was treated conservatively and healed completely. By day 40 post resection, hepatic function was approaching normal parameters. Please refer to Figure 3. Total stay was 73 days in hospital post resection.

Recurrent liver metastases occurred ten months post resection. The Karnofsky performance index [13, 14] at eleven months post resection was 90 out of 100 (100 being best quality of life with no disability). Adrenal and lung metastases noted on follow up computerised tomography appeared fourteen months post resection. Bony metastases were seen at sixteen months post resection. Despite these problems, the patient was still living comfortably at home with her family. The patient died at 18 months post resection from widespread metastatic disease.

### DISCUSSION

Newer techniques of hepatic resection, such as the posterior intra-hepatic approach, have made such surgery safer [15–18]. Consequently, larger resections are being considered for lesions that were previously thought as being unresectable. The limiting step to the quantity resected is the amount of remaining functional liver. Up to seven eights of functional liver parenchyma (that is non-cirrhotic) can be removed [12, 19]. Segment 6 accounts for about 18% of the entire liver weight with the residual liver able to regenerate [11]. The rapidly growing metastatic lesion in this case most likely compromised venous outflow through the superior hepatic veins,

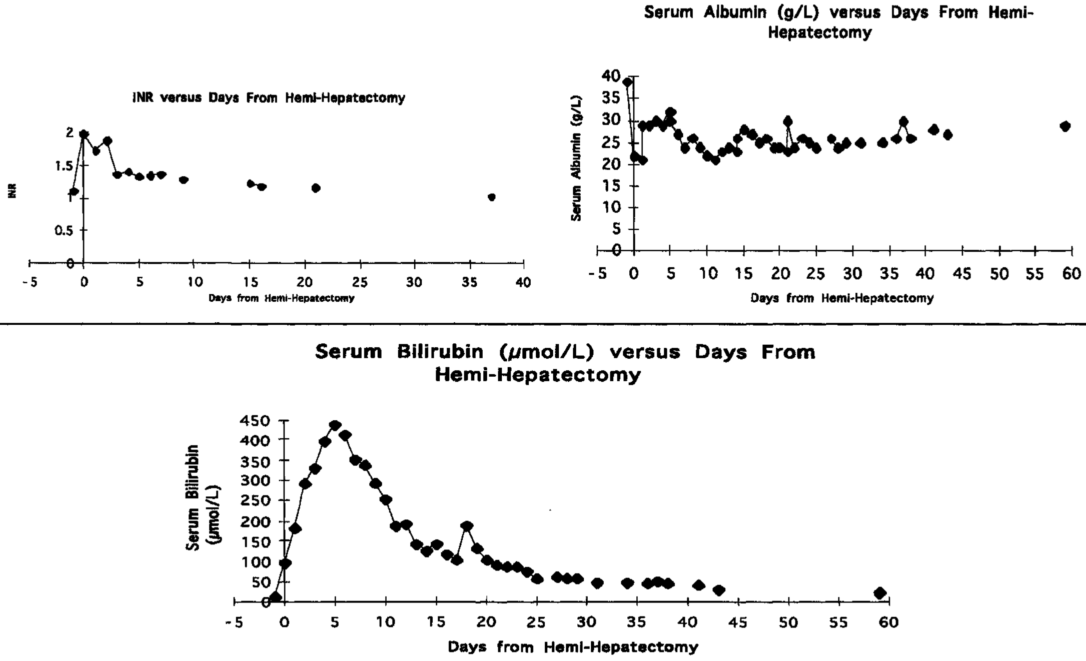

FIGURE 3 Liver function tests. Top left, INR, Top right, serum albumin, bottom, serum bilirubin.

hence the inferior right vein hypertrophied as a collateral channel [20]. If an acute event such as hepatic vein thrombosis (Budd-Chiari syndrome) had occurred in the presence of a normal calibre vein, such compensation is not likely to have taken place. It is possible that the patient already had a larger calibre inferior right hepatic vein. Thus the remnant of the right hepatic lobe was still able to have adequate venous drainage despite the sacrifice of the right hepatic vein. Metastatic disease ultimately killed this patient, not hepatic failure thus the amount of liver resected in the case was compatible with life and an acceptable Karnofsky score post resection. Such a large resection was justified by the fact that the patient had quality time added to her life and there was the possibility of cure. If her metastasis had not been resected, her demise would have been rapid and possibly distressing.

This case illustrates that an 'unresectable' hepatic lesion can be actually resectable if an alternative venous drainage is present. Hence, with lesions of this nature, a pre-operative search for a prominent inferior right hepatic vein by ultrasound, computerised tomography, or even magnetic resonance imaging should be considered.

### *Acknowledgements*

The authors wish to thank the assistance shown by medical records staff at The Queen Elizabeth Hospital.

### *References*

[1] Scheele, J., Stang, R., Altendorf-Hofmann, A. and Paul, M. (1995). Resection of colorectal liver metastases, *World Journal of Surgery*, **19**, 59–71.

[2] Gayowski, T. J., Iwatsuki, S., Madariaga, J. R., Selby, R., Todo, S., Irish, W. and Starzl, T. E. (1994). Experience in hepatic resection for metastatic colorectal cancer: analysis of clinical and pathologic risk factors, *Surgery*, **116**, 703–710.

[3] Adson, M. A. and Van Heerden, J. A. (1980). Major hepatic resections for metastatic colorectal cancer, *Annals of Surgery*, **191**, 576–583.

[4] Keen, W. W. (1899). Report of a case of resection of the liver for the removal of a neoplasm, with a table of seventy-six cases of resection of the liver for hepatic tumours. *Annals of Surgery*, **30**, 267.

[5] Woodington, G. F. and Waugh, J. M. (1963). Results of resection of metastatic tumours of the liver. *American Journal of Surgery*, **105**, 24–9.
[6] Launois, B. and Jamieson, G. G. (1993). *Modern Operative Techniques in Liver Surgery*, 1st edn., pp. 3–36. Melbourne, Edinburgh, London, New York: Churchill Livingstone.
[7] Couinaud, C. (1957). *Le Foie: Études Anatomiques et Chirurgicales*, Paris: Masson et Cie.
[8] Champetier, J., Haouari, H., Le Bas, J.-F., Létoublon, C., Alnaasan, I. and Farah, I. (1993). Large inferior right hepatic vein clinical implications. *Surgical and Radiologic Anatomy*, **15**, 21–9.
[9] Makuuchi, M., Hasegawa, H., Yamazaki, S., Bandai, Y., Watnabe, G. and Ito, T. (1983). The inferior right hepatic vein: ultrasonic demonstration. *Radiology*, **148**, 213–7.
[10] Makuuchi, M., Hasegawa, H., Yamazaki, S. and Takayasu, K. (1987). Four new hepatectomy procedures for resection of the right hepatic vein and preservation of the inferior right hepatic vein. *Surgery Gynecology and Obstetrics*, **164**, 68–72.
[11] Tanaka, K., Nishimura, A., Takenaka, K., Yamada, K., Ishibe, R., Ogata, S., Ishizaki, N. and Taira, A. (1994). Extended central bisegmentectomy – an en bloc resection of hepatic segments 4, 5, 8 and 7: Report of a case. *Surgery Today*, **24**, 170–2.
[12] Baer, H. U., Dennison, A. R., Maddern, G. J. and Blumgart, L. H. (1991). Subtotal hepatectomy: A new procedure based on the inferior right hepatic vein. *British Journal of Surgery*, **78**, 1221–2.
[13] Bonsel, G. J., Essink-Bot, M. L., Klompmaker, I. J. and Sloof, M. J. H. (1992). Assessment of the quality of life before and following liver transplantation. *Transplantation*, **53**, 796–800.
[14] Arbeit, J., Hohn, D. C., Ridge, A. and Spivack, S. D. (1991). Oncology and Cancer Chemotherapy. In: *Current Surgical Diagnosis and Treatment*, 9th edn. Edited by Way, L. W. pp. 1207–46 Sydney: Prentice Hall International.
[15] Launois, B. and Jamieson, G. G. (1992). The Importance of Glisson's capsule and its sheaths in the intra-hepatic approach to resection of the liver. *Surgery Gynecology and Obstetrics*, **174**, 7–10.
[16] Launois, B. and Jamieson, G. G. (1992). The posterior intra-hepatic approach for hepatectomy or removal of segments of the liver. *Surgery Gynecology and Obstetrics*, **174**, 155–8.
[17] Maddern, G. J., Manganas, D. and Launois, B. (1995). Clinical experience with the intra-hepatic posterior approach to the portal triad for right hepatectomy and right segmental resection. *World Journal of Surgery*, **19**, 764–7.
[18] Jamieson, G. G., Corbel, L., Campion, J.-P. and Launois, B. (1993). Resection of anatomical segments of the liver. *Australian and New Zealand Journal of Surgery*, **63**, 251–5.
[19] Guyton, A. C. (1991). Genetic control of protein synthesis, cell function, and cell reproduction. In: *Textbook of Medical Physiology*, Edited by Guyton, A. C., Saunders, W. B. Company, 1991; Ch. 3.
[20] Takayasu, K., Moriyama, N., Muramatsu, Y., Goto, H., Shima, Y., Yamada, T., Makuuchi, M., Yamasaki, S., Hasegawa, H. and Hojo, K. (1985). Intra-hepatic venous collaterals forming *via* the inferior right hepatic vein in 3 patients with obstruction of the inferior vena cava. *Radiology*, **154**, 323–8.

### DRAFT COMMENTARY

The authors describe an extended left hepatic resection with required the removal of all 3 major hepatic veins because of close involvement of tumour. The procedure left the hepatic remnant to drain through the right lower hepatic vein. This type of complex surgery builds on our growing understanding of the intrahepatic anatomical configuration, although the high blood loss and prolonged operations time attests to the complexity of such undertakings.

What is perhaps in question, however is just of how much benefit such procedures will be to individual patients. When extended resections are required for metastatic disease, one is usually dealing with a very large tumour mass and close approximation to major vessels makes the risk of direct invasion or metastatic recurrent spread significant. As a number of studies have shown, tumour recurrence is high under such circumstances and sadly in this case the disease recurred only ten months after liver resection and death occurred in 18 months from recurrent disease.

While clearly extending our repertoire for major surgical procedures, it is likely that such major resections will be undertaken in relatively few patients. As recent surveys have indicated, benefit for liver resection of colorectal metastases is likely to be confined to patients with relatively small tumour mass, replacing less than 25% of the liver volume with less than four lesions which are less than 5, 0 cm in diameter, without neurovascular invasion [1, 3]. Patients presenting with positive lymph nodes and extensive liver involvement with invasion of the main hepatic vessels have very poor survival rates, even after radical resections. The median survival for these patients has been shown to be similar, at around 10 months, to those not referred for surgical resection [2]. While some success is reported with larger single lesions, extended tumour replacement of the liver requiring massive hepatic resection due to metastatic cancer will, all to often, be associated with tumour recorrence or indeed tumour hidden elsewhere. Clearly, very careful evaluation and selection of patients will be required before such major hepatic resections for colorectal cancer and metastases are undertaken and continuing analysis and monitoring of results concerning their survival, morbidity and mortality is essential.

### *References*

[1] Taylor, M., Forster, J., Langer, B., Taylor, B. R., Greig, P. D. and Mahud, C. (1997). A study of prognostic factors for hepatic resection for colorectal metastases. *American Journal of Surgery*, **173**, 467–471.

[2] Jaeck, D., Bachellier, P., Guiget, M., Boudjema, K., Vaillant, J. C., Balladur, P. and Nordlinger, B. and the Association Francaise de Chirurgie (1997) Long-term survival following resection of colorectal hepatic metastases. *British Journal of Surgery*, **84**, 977–980.

[3] Stangl, R., Altendorf-Hoffmann, A., Charnley, R. M. *et al.* (1994). Factors influenciating the natural history of colorectal liver metastases. *Lancet*, **343**, 1405–1410.

Prof. McMaster
University Department of Surgery
University of Birmingham
Queen Elizabeth Hospital
Edgbaston
BIRMINGHAM B12 2TH

